# Analysis of the Role of Homology Arms in Gene-Targeting Vectors in Human Cells

**DOI:** 10.1371/journal.pone.0108236

**Published:** 2014-09-24

**Authors:** Ayako Ishii, Aya Kurosawa, Shinta Saito, Noritaka Adachi

**Affiliations:** 1 Graduate School of Nanobioscience, Yokohama City University, Yokohama, Japan; 2 Advanced Medical Research Center, Yokohama City University, Yokohama, Japan; Michigan State University, United States of America

## Abstract

Random integration of targeting vectors into the genome is the primary obstacle in human somatic cell gene targeting. Non-homologous end-joining (NHEJ), a major pathway for repairing DNA double-strand breaks, is thought to be responsible for most random integration events; however, absence of DNA ligase IV (LIG4), the critical NHEJ ligase, does not significantly reduce random integration frequency of targeting vector in human cells, indicating robust integration events occurring via a LIG4-independent mechanism. To gain insights into the mechanism and robustness of LIG4-independent random integration, we employed various types of targeting vectors to examine their integration frequencies in LIG4-proficient and deficient human cell lines. We find that the integration frequency of targeting vector correlates well with the length of homology arms and with the amount of repetitive DNA sequences, especially SINEs, present in the arms. This correlation was prominent in LIG4-deficient cells, but was also seen in LIG4-proficient cells, thus providing evidence that LIG4-independent random integration occurs frequently even when NHEJ is functionally normal. Our results collectively suggest that random integration frequency of conventional targeting vectors is substantially influenced by homology arms, which typically harbor repetitive DNA sequences that serve to facilitate LIG4-independent random integration in human cells, regardless of the presence or absence of functional NHEJ.

## Introduction

**Figure 1:**
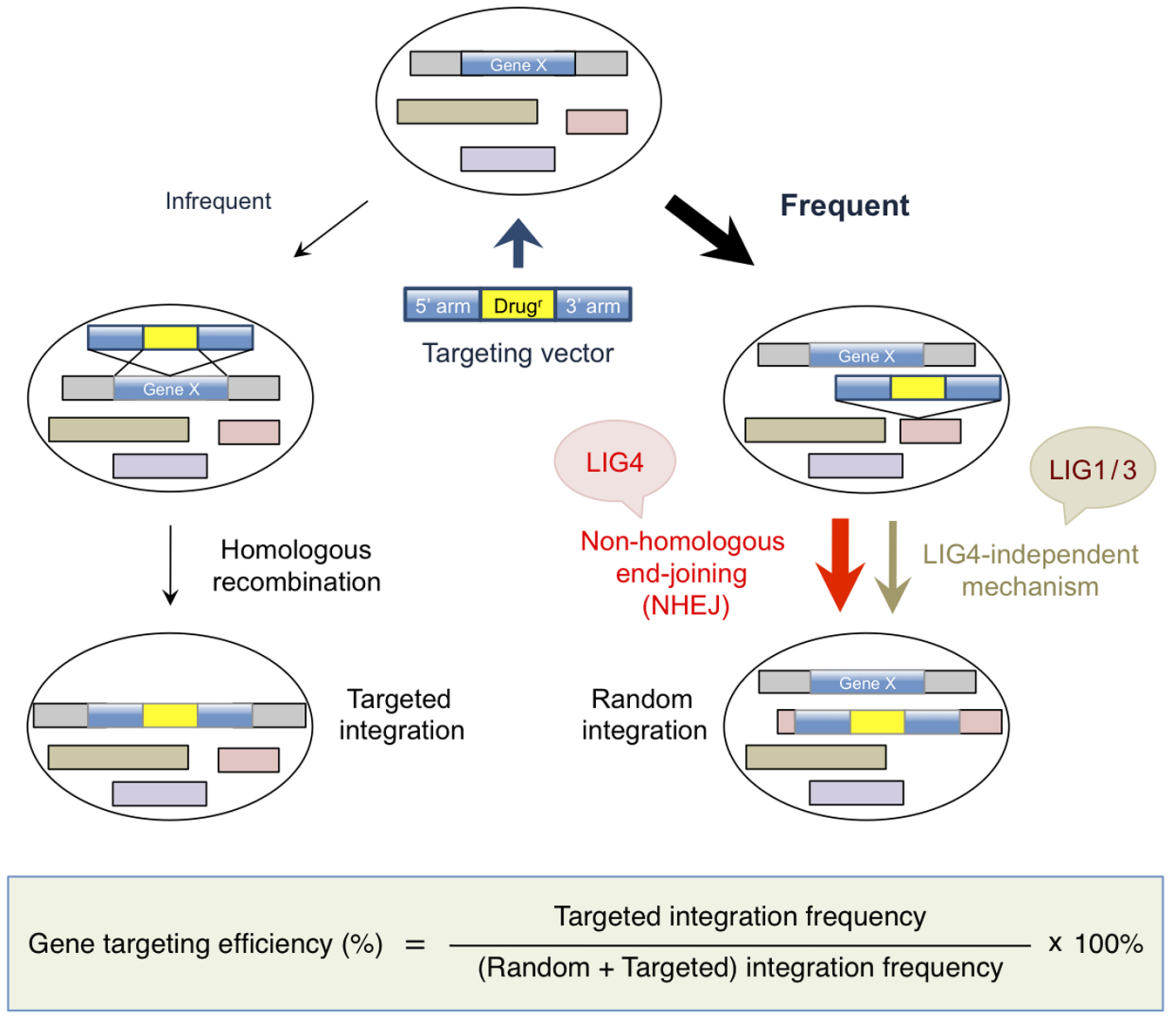
Gene targeting is inefficient in human somatic cells. When targeting vector is transfected into human cells, random integration by non-homologous recombination occurs at least 2 to 3 orders of magnitude more frequently than homologous recombination-mediated targeted integration. The LIG4-dependent NHEJ pathway has been thought to be responsible for random integration, but recent evidence indicates a contribution from LIG4-independent mechanisms that rely on LIG1/3 (DNA ligase I or IIIα). The gene-targeting efficiency is calculated by dividing the number of targeted clones with that of drug-resistant clones analyzed (see Materials and Methods for details).

Gene targeting via homologous recombination provides a powerful means for studying gene function by a reverse genetic approach. In gene-targeting experiments, cells are transfected with targeting vector, which is typically designed and constructed so as to contain a selection marker (drug-resistance) gene flanked with two genomic DNA fragments, called 5'- and 3'-homology arms (or simply 5' and 3' arms, or left and right arms) [1]. After transfection, these two arms should be homologously recombined with the target genome sequence in the cell to achieve successful genetic modification of the chromosomal locus [2]. In human somatic cells, the frequency of such targeted integration is at least two to three orders of magnitude lower than that of random integration [3] (depicted in Figure 1). It therefore seems reasonable to expect that reducing random integration events would enhance gene targeting by increasing the ratio of targeted to random integration.

Non-homologous end-joining (NHEJ), which repairs DNA double-strand breaks (DSBs) in a Ku-dependent manner [4], is responsible for nearly all random integration events in lower eukaryotes, such as *Neurospora crassa*, and thus NHEJ deficiency dramatically enhances gene targeting [5], [6]. Unfortunately, however, this is not the case for human somatic cells, as apparently NHEJ is not the sole mechanism of random integration [7], [8] (Figure 1). Although earlier studies have suggested a substantial role of NHEJ in random integration [9], [10], we have previously observed robust random integration events in cells lacking DNA ligase IV (LIG4, a critical NHEJ factor [11]). This finding strongly suggests the involvement of other DSB repair pathways, most presumably alternative end-joining, in causing random integration [7]. The molecular mechanism of alternative end-joining in DSB repair is not yet fully understood; however, recent work has established that alternative end-joining is mechanistically distinct from NHEJ (i.e., Ku/LIG4-dependent NHEJ) [12], [13] and requires DNA ligase I or III, not LIG4, in repair of DSBs [14]-[16]. Additionally, it is now accepted that initiation of alternative end-joining requires end resection of the broken DNA to produce single-stranded DNA that is used for strand annealing, a mechanism similar to initiation of homologous recombination involving single-strand annealing (SSA) [17], [18]. By virtue of this mechanism, alternative end-joining is believed to favor microhomologies (≥4 nt) for the joining of DNA ends, unlike NHEJ that typically joins DNA ends with short or no homology (0–4 nt) [4], [19], [20]. However, there exists an alternative end-joining mechanism with no apparent microhomologies [21], [22].

Despite its biological and medical importance, the precise mechanism of random integration in human somatic cells remains largely unclear, and at least two distinct mechanisms exist — LIG4-dependent NHEJ and LIG4-independent alternative end-joining. Recently, Suzuki *et al*. [23] have reported that chromosomal integration of plasmid DNA (a non-targeting vector) transfected into mouse embryonic stem (ES) cells is mostly a complex reaction with frequent terminal deletions of the plasmid and the genome. Interestingly, sequence analysis of those random integrants revealed a frequent use of microhomologies at the plasmid-genome junctions [23]. This finding may suggest the occurrence of random integration events via alternative end-joining, given the aforementioned microhomology preference of this mechanism in repair of DSBs. However, the view that NHEJ avoids using microhomologies may not be entirely correct [19]. In addition, mouse ES cells express low levels of DNA-PKcs and thus may not be fully competent for NHEJ [24]. Hence, it is yet uncertain whether alternative end-joining is involved in random integration events occurring in human cells with normal NHEJ capacity.

The human genome, unlike the genomes of lower eukaryotes, is large in size (3×10^9^ bp) and contains a huge amount of repetitive DNA sequences; among these, short interspersed nucleotide elements (SINE) such as Alu and long interspersed nucleotide elements (LINE) occur in ∼1–2×10^6^ copies per genome [25], [26]. Thus, SINE and LINE sequences occupy 36% of the human genome. The intact human SINEs and LINEs are ∼300 bp and 6 kb, respectively, but some SINEs and most LINEs are fragments and can be as short as 100 bp or less. As mentioned above, conventional gene-targeting vectors possess two homology arms whose DNA sequence is identical to the target genome sequence. In designing and constructing a targeting vector, it is generally unavoidable to incorporate a repetitive DNA sequence(s) into homology arms (especially when one intends to make long arms) because of the high abundance of repetitive DNA fragments in the genome. Thus, most if not all targeting vectors contain a repetitive DNA fragment(s) in their homology arms.

We have previously shown that although LIG4-deficient human cells exhibit reduced integration frequencies when transfected with non-targeting vectors having no homology to the host genome, such a decrease was not observed when targeting vectors were employed [7]. This suggests that homology arms present in the vector somehow facilitated random integration in a LIG4-independent fashion. In this study, we generated various types of human *HPRT* targeting vectors to analyze the relationship between the frequency of LIG4-dependent and LIG4-independent random integration and the length (or presence) of homology arms. For this purpose, we employed the human pre-B leukemia cell line Nalm-6 (NHEJ-competent) and its *LIG4*-null (NHEJ-deficient) cells. Additionally, using these cell lines and targeting vectors for more than ten different human genes, we performed a detailed analysis on random integration frequency and homology arms or repetitive DNA sequences. Our data collectively suggest that integration frequency of targeting vector correlates well with the lengths of homology arms and repetitive DNA sequences, which likely facilitate alternative end-joining-mediated random integration even in the presence of functional NHEJ.

## Results and Discussion

### Long homology arms stimulate LIG4-independent random integration of the targeting vector

**Figure 2:**
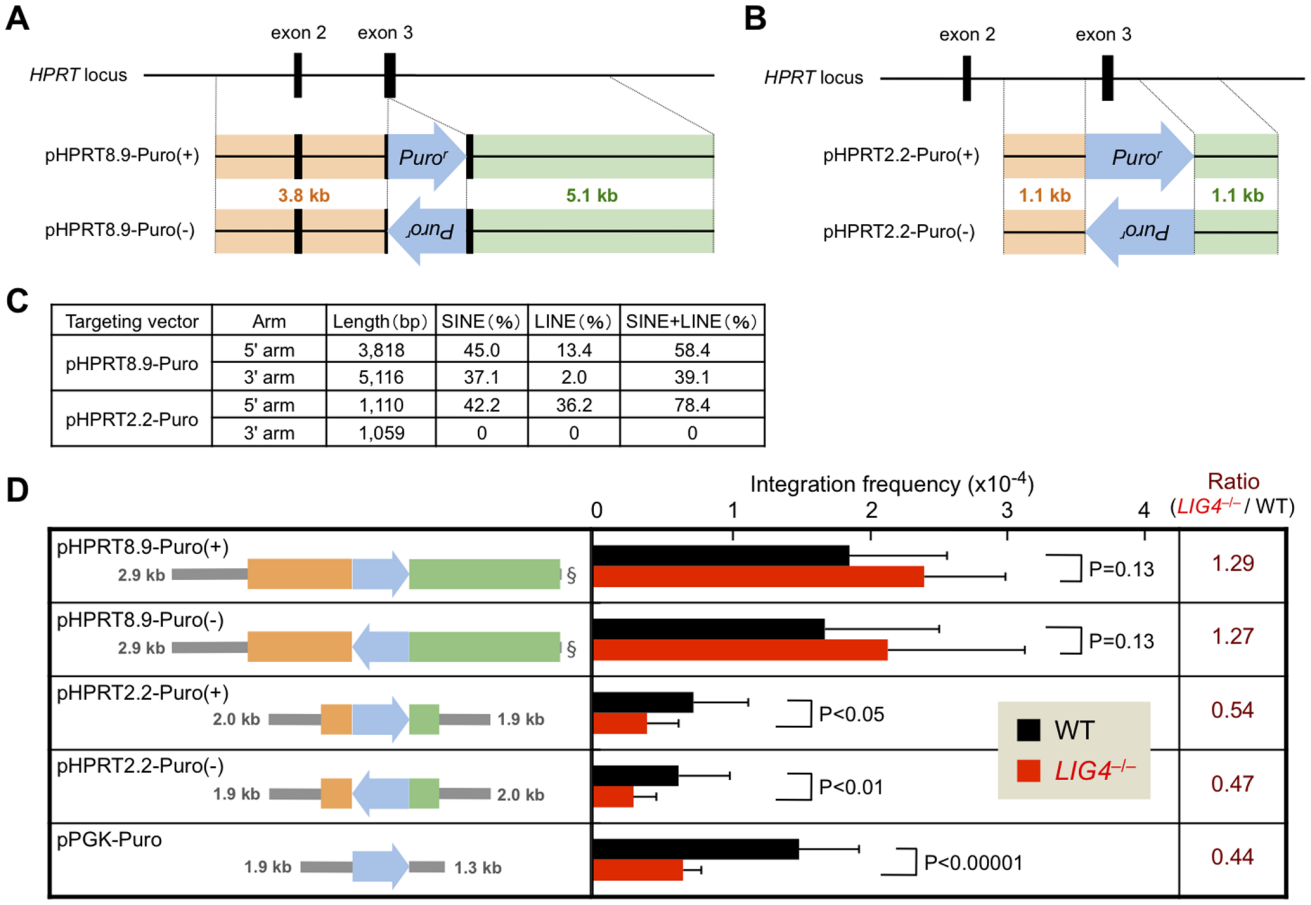
*HPRT* targeting vectors with long, but not short, homology arms stimulate NHEJ-independent random integration. (A) Schematic representation of *HPRT* targeting vectors pHPRT8.9-Puro(+) and pHPRT8.9-Puro(−). (B) Schematic representation of *HPRT* targeting vectors pHPRT2.2-Puro(+) and pHPRT2.2-Puro(−). (C) Structural features of the *HPRT* targeting vectors. (D) Integration frequency of *HPRT* targeting vectors and pPGK-Puro (a non-targeting vector) in human Nalm-6 wild-type and *LIG4*-null cells. The ratio of integration frequency in *LIG4*-null to wild-type cells is indicated in the right column. At least six independent experiments were performed for each vector. Note that pPGK-Puro harbors little or no homology to the human genome. Grey lines indicate the lengths of plasmid backbones, and § denotes a 14-bp sequence.

We first examined the integration frequency of four *HPRT* targeting vectors pHPRT8.9-Puro(+), pHPRT8.9-Puro(−), pHPRT2.2-Puro(+) and pHPRT2.2-Puro(−), and pPGK-Puro (a non-targeting vector with no homology arms) in Nalm-6 wild-type and *LIG4*-null cells [27]. As shown in Figure 2A and B, these targeting vectors were designed to disrupt exon 3 of the *HPRT* gene. The pHPRT8.9-Puro vectors have a 3.8-kb 5' arm and a 5.1-kb 3' arm (the two arms are adjacent to one another in the genome), whereas the pHPRT2.2-Puro vectors have shorter homology arms (1.1 kb each; located 0.7 kb apart in the genome) (Figure 2A–C and Figure S1 in File S1). In these vectors, 5' and 3' arms flank a drug-resistance gene cassette (*Puro^r^*) present in forward (+) or reverse (−) orientation to the gene. As shown in Figure 2D, integration frequency of pPGK-Puro was significantly lower in *LIG4*-null cells than in wild-type cells (P<0.00001; n = 11), thus confirming the contribution of NHEJ to random integration. Very similar results were obtained with vectors containing other drug-resistance genes (data not shown; [7]). In contrast, integration frequency of pHPRT8.9-Puro(+) and pHPRT8.9-Puro(−) was not decreased, but rather slightly increased in *LIG4*-null cells (Figure 2D). Interestingly, vectors with shorter homology arms, pHPRT2.2-Puro(+) and pHPRT2.2-Puro(−), displayed decreased integration frequency in *LIG4*-null cells (P<0.05 for pHPRT2.2-Puro(+) and P<0.01 for pHPRT2.2-Puro(-); n = 8), a result similar to that of non-targeting vectors. These results suggest that the presence of long homology arms serves to facilitate LIG4-independent random integration of the targeting vector.

**Figure 3:**
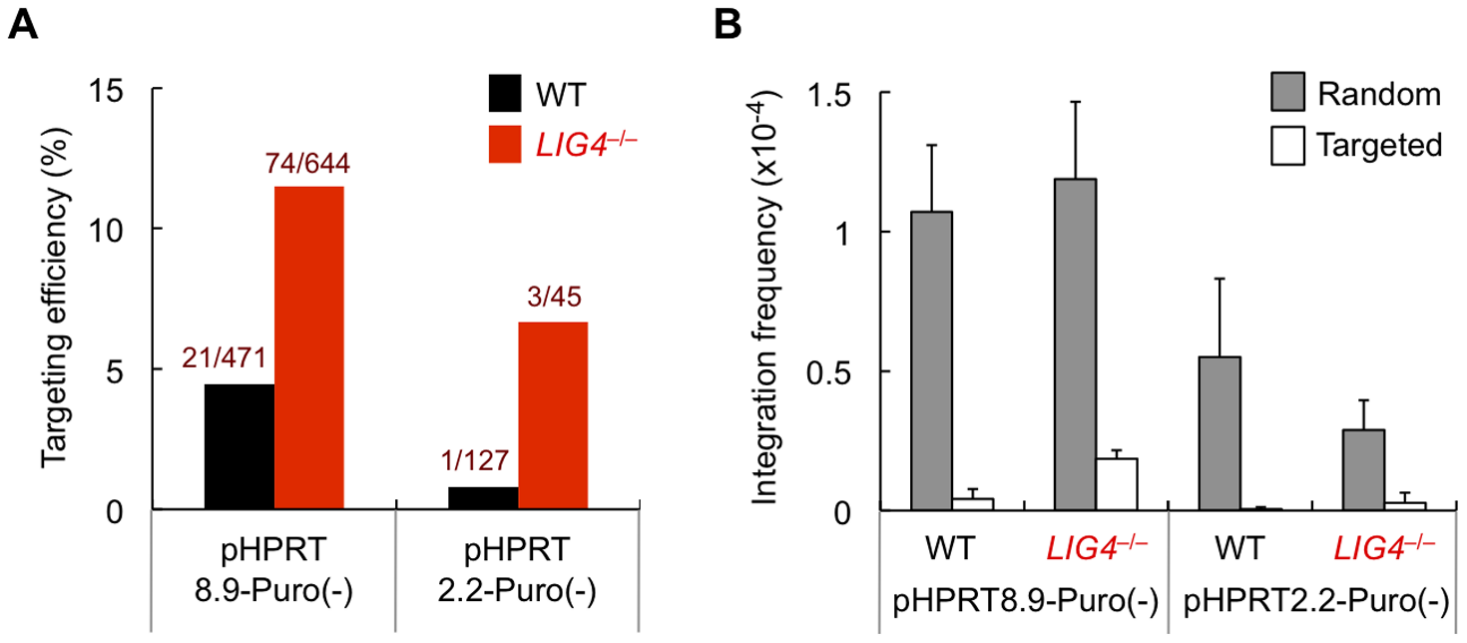
The short-arm vector pHPRT2.2-Puro(−) functions as a genuine targeting vector. (A) Gene-targeting efficiency of pHPRT8.9-Puro(−) and pHPRT2.2-Puro(−) in wild-type and *LIG4*-null cells. (B) Random and targeted integration frequencies of pHPRT8.9-Puro(−) and pHPRT2.2-Puro(−) in wild-type and *LIG4*-null cells. At least three independent experiments were performed for each vector.

As the pHPRT2.2-Puro vectors harbor relatively short arms, we next examined whether pHPRT2.2-Puro(−) functioned as a genuine targeting vector. We thus picked puromycin-resistant colonies derived from pHPRT2.2-Puro(−)-transfected cells and confirmed that correctly targeted clones are actually obtained (1 out of 127 clones in wild-type cells and 3 out of 45 clones in *LIG4*-null cells) (Figure 3A). The increased targeting efficiency associated with *LIG4* deficiency is consistent with previous studies using human cells [7], [28]. Of note, although pHPRT2.2-Puro(−) exhibited low targeting efficiencies compared to pHPRT8.9-Puro(-), gene-targeting enhancement associated with the *LIG4* deficiency was more pronounced for pHPRT2.2-Puro(−) (∼10-fold increase) than for pHPRT8.9-Puro(−) (∼2–3-fold increase)(Figure 3A). Consistent with the aforementioned data, random integration frequency of pHPRT2.2-Puro(−) was reduced in *LIG4*-null cells, while that of pHPRT8.9-Puro(−) was not (Figure 3B).

### LIG4-independent random integration is decreased when a homology arm with repetitive DNA sequences is deleted from the targeting vector

**Figure 4:**
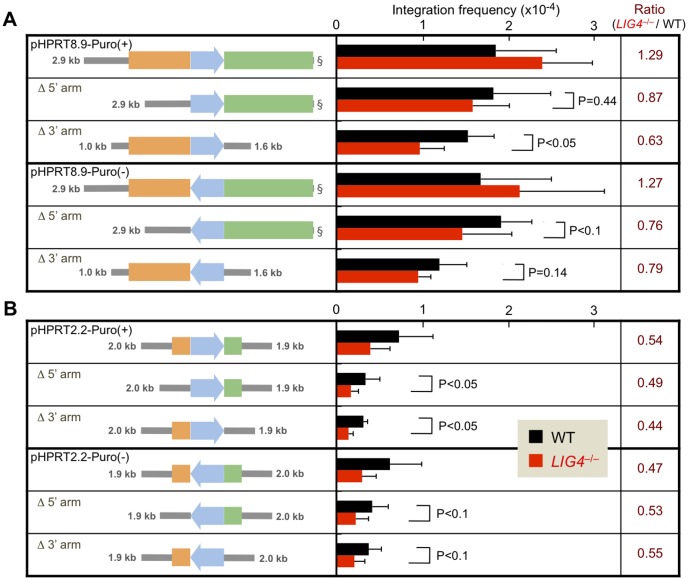
NHEJ-independent random integration is significantly decreased when a homology arm is deleted from the *HPRT* targeting vector. (A) Integration frequency of pHPRT8.9-Puro vectors and their derivatives in Nalm-6 wild-type and *LIG4*-null cells. The ratio of integration frequency in *LIG4*-null to wild-type cells is indicated in the right column. At least five independent experiments were performed for each vector. The data for the arm-proficient vectors are the same as that in Figure 2D. (B) Integration frequency of pHPRT2.2-Puro vectors and their derivatives in Nalm-6 wild-type and *LIG4*-null cells. The ratio of integration frequency in *LIG4*-null to wild-type cells is indicated in the right column. At least three independent experiments were performed for each vector. Symbols are as in Figure 2D, and the data for the arm-proficient vectors are the same as that in Figure 2D.

To further investigate the relationship between the presence/length of homology arms and the frequency of LIG4-independent random integration, we then generated "imperfect" targeting vectors lacking either a 5’ or 3’ arm by using the four types of *HPRT* targeting vectors described above. Each vector was transfected into Nalm-6 wild-type and *LIG4*-null cells to calculate the integration frequency and the ratio between the two cell lines. As shown in Figure 4A, the absence of either arm significantly decreased the integration frequency of pHPRT8.9-Puro(+) and pHPRT8.9-Puro(−) in *LIG4*-null cells relative to wild-type cells. In contrast, deleting either arm of pHPRT2.2-Puro(+) and pHPRT2.2-Puro(−) had a marginal effect on the ratio of integration frequency in *LIG4*-null to wild-type cells (Figure 4B). These results suggest that integration frequency in *LIG4*-null cells is roughly proportional to the total length of homology arms present in the vector, further supporting the notion that homology arms facilitate LIG4-independent targeting-vector integration.

It is interesting to note that our results also suggest that the direction or position of the drug-resistance gene relative to the homology arm may affect LIG4-independent random integration. In the experiments using pHPRT8.9-Puro-derived imperfect vectors, the absence of an arm located downstream of *Puro^r^* (i.e., the 5' arm for (−) vectors and the 3' arm for (+) vectors) had a greater impact on LIG4-independent random integration (i.e., the ratio of integration frequency in *LIG4*-null to wild-type cells). Likewise, in the experiments using pHPRT2.2-Puro-derived imperfect vectors, the impact of 5'-arm deletion on LIG4-independent random integration was slightly more prominent when the 5' arm was originally located downstream of *Puro^r^*. This position effect, however, was not observed in 3' arm-deleted pHPRT2.2-Puro vectors. It should be mentioned that the pHPRT2.2-Puro 3' arm harbors essentially no repetitive sequences, whereas the 5' arm contains a large number of SINE/LINE sequences (78.4% occupancy)(see Figure 2C; in pHPRT8.9-Puro vectors, the two arms similarly contain repetitive sequences). Together, these findings imply that repetitive DNA sequences present in the homology arms facilitate LIG4-independent targeting-vector integration, especially when these repetitive sequences are present downstream of the drug-resistance gene cassette, which has a promoter. Although the precise mechanism of this possible position effect is currently unclear, we speculate that transcription of the marker gene (i.e., transient expression occurring before or during vector DNA integration) may lead to a partially denatured state of the downstream region and then a denatured repetitive sequence(s) present in the arm could serve to facilitate LIG4-independent integration of the vector into the genome.

### Integration frequency of targeting vector correlates with the lengths of homology arms and repetitive DNA sequences regardless of NHEJ status

The above results using various types of *HPRT* vectors suggest a correlation between the length of arms and the absolute frequency of random integration. Importantly, this correlation was observed in wild-type cells, though less prominent than in *LIG4*-null cells; for instance, the integration frequency of pHPRT8.9-Puro(−) was ∼2-fold higher than that of pHPRT2.2-Puro(−)(Figure 3B), suggesting that LIG4-independent random integration does occur in cells with normal NHEJ function. To test this directly, we knocked down the expression of DNA ligase I or IIIα in wild-type and *LIG4*-null cells. Because the LIG4-independent integration mechanism does not rely on LIG4, either or both of DNA ligase I and IIIα should be involved in this mechanism. As shown in Figure S2 in File S1, transfection of *LIG1* siRNA or *LIG3* siRNA had an effect on reducing random integration frequency in wild-type cells, even though the siRNA-mediated knockdown was somewhat incomplete. These results suggest that DNA ligase I and IIIα are both involved in LIG4-independent random integration events, consistent with recent findings on DNA ligase usage in DSB repair [14]–[16].

**Figure 5:**
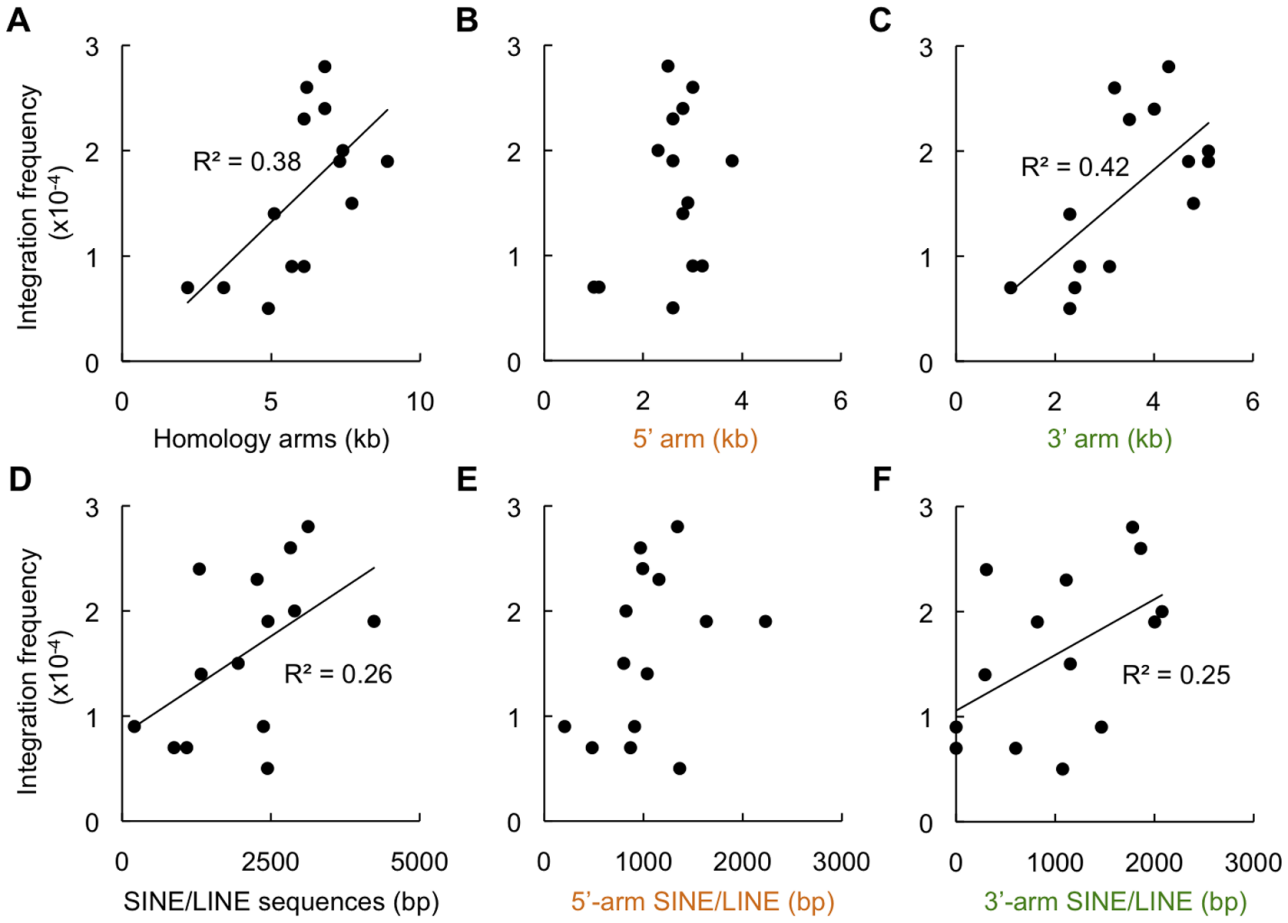
Integration frequency of targeting vector correlates with the lengths of homology arms and repetitive DNA sequences. Integration frequencies of pHPRT8.9-Puro(+), pHPRT2.2-Puro(+), and twelve other (non-*HPRT*) gene-targeting vectors are shown as a function of the total length of homology arms (A), 5’-arm length (B), 3’-arm length (C), the total length of SINE/LINE sequences present in the arms (D), 5’-arm SINE/LINE length (E), and 3’-arm SINE/LINE length (F). The R^2^ values shown in the graphs were calculated from the fourteen points. See also Figures S4 and S5 in File S1 for details.

We next performed a comprehensive analysis using various gene-targeting vectors to examine whether the correlation between arm length and integration frequency is actually observed in wild-type cells. For this purpose, we used twelve different gene-targeting vectors (Figure S3 in File S1). In these vectors, the lengths of 3' arms vary, while 5' arms of most of the vectors are roughly similar in size (∼2.5–3.2 kb) but contain different lengths of repetitive DNA sequences (i.e., SINE and LINE sequences), and drug-resistance gene cassettes are placed in the same (forward) direction. From the observations described above, we predicted that the absolute integration frequency would be proportional to the length of the 3' arm (or both arms) and also to the total length of repetitive DNA sequences present in the vector. As shown in Figure 5, this was indeed the case. We found that the length of arms, especially that of 3’ arm, had a positive, albeit weak, correlation with the integration frequency in wild-type cells (total arm length, R^2^ = 0.38; 3’-arm length, R^2^ = 0.42)(Figure 5A–C). We also found a weak correlation between the integration frequency and the length of SINE/LINE sequences present in the arms (total SINE/LINE length, R^2^ = 0.26; 3’-arm SINE/LINE length, R^2^ = 0.25)(Figure 5D–F). Since this correlation was not fully statistically significant and seemed a bit lower than we expected, we analyzed the characteristics of those targeting vectors whose integration frequency was deviating largely from the approximation. This analysis led us to notice that vectors containing an extremely short 3’ arm (*ARTEMIS* and *APTX*) conferred low integration frequencies, whereas a vector with a relatively long (∼0.6 kb) LINE fragment in the distal end of the 5’ arm (*RAG1*) gave a high integration frequency. When these "exceptional" vectors were excluded from the data set to redraw a fitted curve, a stronger correlation (R^2^ = 0.47) was observed between the total SINE/LINE length and the integration frequency (Figure S5 in File S1). Even though this R^2^ value is still not high enough to show a statistical significance, our results collectively suggest that the length of repetitive sequences within arms as well as arm length may be a major determinant of the integration frequency of targeting vectors.

We then examined which interspersed element, SINE or LINE, contributed to facilitating random integration. As shown in Figure S4 in File S1, the length of SINE sequences had roughly the same impact on integration frequency as the length of SINE/LINE sequences. In contrast, the length of LINE sequences did not appear to have a clear correlation with the integration frequency. Previous work has shown, however, that human LINEs contribute to chromosomal integration of exogenous DNA [29]. Indeed, we observed that the *RAG1* targeting vector with a 579-bp LINE fragment in its short 5' arm, showed a higher integration frequency than expected as described above. In the human genome, the copy number of SINEs is higher than LINEs [30], and thus SINE-containing sequences may gain easier access to the genome sequence. Moreover, SINEs tend to distribute in gene-rich GC-rich regions, while LINEs in gene-poor AT-rich regions [30]. In this regard, recent work has reported that integration of exogenous DNA into mouse ES cell chromosomes shows preference into genes [23]. Alternatively or additionally, as LINEs present in the targeting vectors are short fragments (<300 bp)(Figure S1 in File S1), these LINE fragments may lack the ability to facilitate random integration.

**Figure 6:**
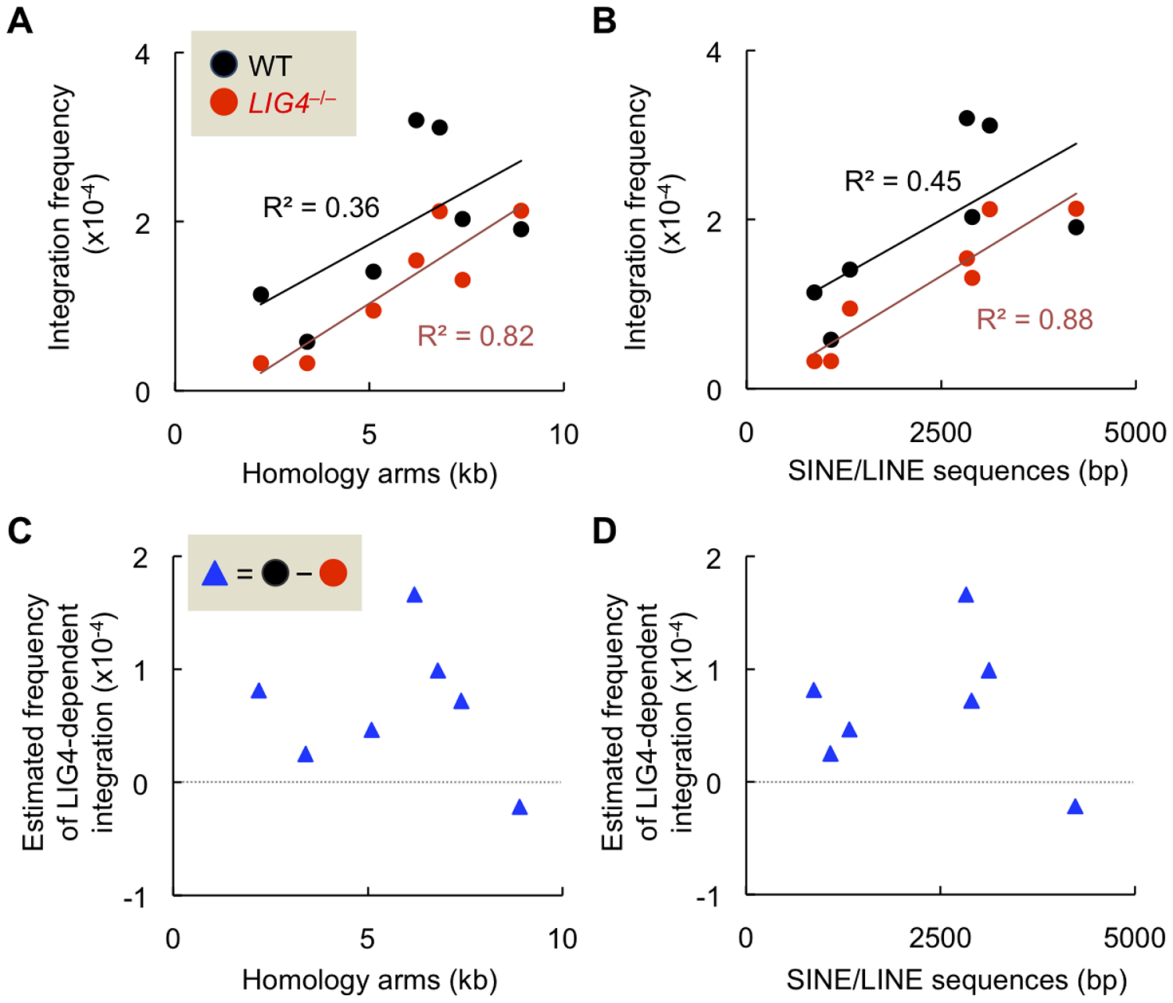
Integration frequency of targeting vector correlates well with the lengths of homology arms and repetitive DNA sequences, particularly in the absence of LIG4. (A, B) Integration frequencies of seven gene-targeting vectors in wild-type and *LIG4*-null cells are shown as a function of the total length of homology arms (A) and the total length of SINE/LINE sequences present in the arms (B). (C, D) Estimated frequencies of LIG4-dependent integration are shown as a function of the total length of homology arms (C) and the total length of SINE/LINE sequences present in the arms (D). The values were calculated by subtracting the integration frequency in *LIG4*-null cells from that in wild-type cells. See text for more details.

Finally, we set out to directly compare the frequencies of LIG4-dependent and independent random integration in wild-type and *LIG4*-null cells by using seven different gene-targeting vectors. For this purpose, we subtracted the integration frequency in *LIG4*-null cells from that in wild-type cells to estimate the frequency of LIG4-dependent integration. The subtracted values are only an approximation, but should reflect the frequency of LIG4-dependent integration, although likely underestimated, given that the LIG4-independent mechanism may not be fully active in NHEJ-competent cells. As shown in Figure 6A and B, the integration frequency in *LIG4*-null cells was directly proportional to the arm length and the amount of repetitive sequence (R^2^ = 0.82 and R^2^ = 0.88, respectively), indicating a statistically significant correlation between LIG4-independent random integration frequency and the length of homology arms or repetitive sequences of the targeting vector. Importantly, a similar, but less pronounced, correlation was also found in wild-type cells (R^2^ = 0.36 and R^2^ = 0.45, respectively), consistent with the aforementioned data shown in Figure 5. In sharp contrast, the subtracted values had no obvious correlation with the lengths of homology arms or repetitive DNA sequences, as shown in Figure 6C and D. These data provide further evidence that LIG4-independent integration does occur when NHEJ is functionally normal, and that LIG4-independent random integration, but not LIG4-dependent random integration, is substantially affected by homology arms and repetitive sequences of the targeting vector.

As the mechanism of random integration remains largely unclear, how homology arms with repetitive sequences facilitate targeting-vector random integration is even more enigmatic. Almost undoubtedly, random integration of non-targeting vectors in *LIG4*-null cells is mediated by alternative end-joining, as the involvement of NHEJ or SSA is highly unlikely (and this is also true for joining at the arm-deleted side of aforementioned *HPRT* vectors). When a homology arm(s) are present, an increase in integration frequency was observed in our experiments. This increase most likely depends on alternative end-joining, given that the presence of SINE/LINE fragments increases the amount of sequences with microhomology to the genome. The possibility of SSA involvement cannot be fully excluded, however, as most SINE/LINE fragments have enough length of homology to carry out homologous recombination, even though perfect homology may not be available due to the heterogeneity of these repetitive sequences [26]. Indeed, recent work by Escribano-Díaz *et al*. showed that knockdown of 53BP1/RIF1 (which act to stimulate NHEJ) led to a very similar increase in alternative end-joining and SSA at I-*Sce*I-induced DSBs [31]. Importantly, for SSA or microhomology-mediated alternative end-joining to bring about random integration, not only must the end of the vector be extensively deleted, but also the target genome should have a pre-existing DSB at or near a SINE/LINE fragment. This is likely enough, given the observed frequent terminal deletions of transfected plasmid [23] and the high abundance of SINE/LINE fragments in the genome. However, a more likely possibility for LIG4-independent integration may be a synthesis-dependent microhomology-mediated end-joining (SD-MMEJ) mechanism, as proposed by Yu and McVey [22]. This model well explains frequent terminal modifications observed at plasmid-genome junctions in random integrants from mouse ES cells [23] as well as little or no apparent junctional microhomologies in *LIG4*-null cells ([21]; our unpublished observations).

### Concluding remarks

In this paper, we have shown, to our knowledge for the first time, that targeting vectors with long homology arms tend to confer high random-integration frequencies in human cells, most likely by virtue of the presence of repetitive DNA sequences. Perhaps conflicting with this finding, it is generally accepted that lengthening homology arms is beneficial for increasing gene-targeting efficiency [3], [32]. Indeed, the targeting efficiency of pHPRT8.9-Puro(−) was greater than that of pHPRT2.2-Puro(−) in our experiments (Figure 3A). It should be noted, however, that pHPRT8.9-Puro(-) conferred higher random and targeted integration frequencies than did pHPRT2.2-Puro(−) (Figure 3B). Additionally, we examined and compared the targeting efficiency in Nalm-6 cells using more than ten different gene-targeting vectors, and did not observe a clear correlation between the arm length and the targeting efficiency (Figure S6 in File S1), although this could simply be due to the unavoidable lack of uniformity of the targeting constructs used in those experiments. It is therefore suggested that the length of homology arms affects both random and targeted integration, and hence the presence of long homology arms may allow for enhanced targeting efficiency but simultaneously increase the absolute frequency of random integration. We have also shown in this study that LIG4-independent end-joining mechanisms contribute to random integration events, even when NHEJ is functionally normal, and that repetitive DNA sequences, especially SINEs, present in targeting-vector arms serve to facilitate LIG4-independent random integration. These results imply that constructing a targeting vector that contains no repetitive DNA sequence should be a promising way to minimize random integrants. This may particularly be the case when an artificial nuclease-based gene targeting system, such as TALEN or CRISPR, is being employed, in which a targeting vector does not require long homology arms. Even more importantly, the identification of a factor(s) specifically involved in LIG4-independent integration mechanisms will be of great value for enhancing gene targeting, assuming that simultaneous suppression of LIG4-dependent and independent mechanisms would have a dramatic effect on reducing random integrants and if this strategy does not disturb viability or genome integrity of human somatic cells.

## Materials and Methods

### Vectors

The long-arm *HPRT* targeting vectors pHPRT8.9-Puro(+) and pHPRT8.9-Puro(−) were constructed as previously described [7]. Other gene-targeting vectors, including the short-arm *HPRT* targeting vectors pHPRT2.2-Puro(+) and pHPRT2.2-Puro(−), were constructed using the MultiSite Gateway system (Life Technologies, Carlsbad, CA) to assemble two homology arms and a drug-resistance gene cassette, as previously described [1], [27]. Genomic fragments for homology arms were PCR amplified using Nalm-6 genomic DNA as template with primers listed in Figures S7 and S8 in File S1. Imperfect vectors lacking either arm of pHPRT8.9-Puro(+) and pHPRT8.9-Puro(−) were constructed as shown in Figure S9 in File S1. Imperfect vectors lacking either arm of pHPRT2.2-Puro(+) and pHPRT2.2-Puro(−) were constructed by digestion with SacI (for 5'-arm deletion) or SalI (for 3'-arm deletion), followed by self-ligation of the linearized plasmid DNA. All the plasmid vectors were purified with Qiagen Plasmid Maxi Kits (Qiagen K.K., Tokyo) and linearized with an appropriate restriction enzyme prior to transfection [1].

### Cell Culture

The human pre-B leukemia cell line Nalm-6 [21] and its derivative (the *LIG4*−/− cell line; [27]) were maintained in a 5% CO_2_ incubator at 37°C in ES medium (Nissui Seiyaku Co., Tokyo, Japan) supplemented with 10% calf serum (Hyclone, Logan, UT) and 50 µM 2-mercaptoethanol. The *LIG4^−/−^* cells were generated as described [27].

### Integration Assays and Gene-targeting Experiments

Transfection of vector DNA or siRNA was performed as described previously [1],[7],[33]. For integration assays, 4×10^6^ cells were electroporated with 4 µg of linearized plasmid DNA, cultured for 22 hr, and replated at a density of 0.5–1×10^6^ cells per 90-mm dish into agarose medium containing 0.5 µg/ml puromycin (Wako Pure Chemical, Osaka, Japan). Meanwhile, small aliquots of the transfected cells were replated into drug-free agarose medium to determine the plating efficiency. After cultivation for 2–3 weeks, the resulting colonies were counted, and the total integration frequency was calculated by dividing the number of drug-resistant colonies with that of surviving cells. For gene-targeting experiments, each targeting vector was linearized and transfected into wild-type or *LIG4^−/−^* cells. After a 2–3 week incubation, genomic DNA was prepared from drug-resistant colonies and subjected to PCR and Southern blot analysis as described [7]. The gene-targeting efficiency was calculated by dividing the number of targeted clones with that of drug-resistant clones analyzed (Figure 1). The targeted integration frequency was calculated by multiplying the total integration frequency by the targeting efficiency. The random integration frequency was calculated by subtracting the targeted integration frequency from the total integration frequency.

## Supporting Information

File S1 **Figures S1-S9.** Figure S1. Schematic representation of repetitive DNA sequences present in the *HPRT* vectors used in this study. The location and length (bp) of each SINE/LINE fragment is based on the UCSC Genome Browser Database: Update 2006 (Nucleic Acids Res. 34:D590–D598, 2006). Figure S2. Impact of siRNA-mediated knockdown of DNA ligase I or IIIα on integration frequency. (A) The nucleotide sequence of *LIG1* and *LIG3* siRNA. These siRNAs were designed as reported previously (Nucleic Acids Res. 36: 3297–3310, 2008). (B) Western blot analysis for DNA ligase I and IIIα in siRNA-transfected Nalm-6 wild-type and *LIG4*-null cells. M, mock-transfected. (C, D) Integration frequency in wild-type and *LIG4*-null cells treated with *LIG1* siRNA (C) or *LIG3* siRNA (D). A non-targeting vector (pβactin-His; Nucleic Acids Res. 36: 6333–6342, 2008) was used for transfection. The integration frequency in mock-transfected wild-type cells was taken as 1, and the relative integration frequency was calculated. Figure S3. Structural features of gene-targeting vectors used for the analysis of integration frequency. (A) Fundamental structure of targeting vectors. In all the vectors, 5' and 3' arms flank the drug-resistance gene cassette (*Puro^r^*), which is placed in the forward direction. (B) Structural features of the fourteen gene-targeting vectors used. Shown are the lengths of 5' and 3' arms and SINE/LINE sequences within each arm and the integration frequency. The length of SINE/LINE is based on the UCSC Genome Browser Database: Update 2006 (Nucleic Acids Res. 34:D590–D598, 2006). Figure S4. Integration frequency of targeting vector as a function of the length of repetitive DNA sequences. Integration frequencies of pHPRT8.9-Puro(+), pHPRT2.2-Puro(+), and twelve other gene-targeting vectors are shown as a function of the total length of SINE sequence (A), 5’-arm SINE length (B), 3’-arm SINE length (C), the total length of LINE sequence (D), 5’-arm LINE length (E), and 3’-arm LINE length (F). See also Figure 5. Figure S5. Correlation between the integration frequency and repetitive DNA sequences. (A) Integration frequencies of pHPRT8.9-Puro(+), pHPRT2.2-Puro(+), and twelve other gene-targeting vectors as a function of the total length of SINE/LINE sequences. Note that this graph is the same as Figure 5D. (B) Same as (A), except that the three vectors have been omitted from the data set. Note that the redrawn fitted curve reveals a stronger correlation between the total SINE/LINE length and the integration frequency. See text and Figure 5 for details. Figure S6. Gene-targeting efficiency is little affected by the length of homology arms. Targeting efficiencies are shown as a function of the total length of homology arms of the targeting vector. The twelve non-*HPRT* gene-targeting vectors were used for the analysis (see Figure S3B in File S1). Figure S7. PCR primers used to amplify the homology arms of pHPRT2.2-Puro vectors. The restriction sites used to construct the arm-deleted vectors are shown in red (SacI) or blue (SalI). Figure S8. PCR primers used to amplify the homology arms of non-*HPRT* targeting vectors. Red denotes *attB* sequences. Figure S9. Schematic representation of construction of imperfect pHPRT8.9-Puro vectors lacking the 3' arm (A) or 5' arm (B).
(PDF)
