# Supplementary material for: Analysis of the Role of Homology Arms in Gene-Targeting Vectors in Human Cells
Source: PLoS One. 2014 Sep 24;9(9):e108236. doi: 10.1371/journal.pone.0108236 (PMC4176728; doi:10.1371/journal.pone.0108236)
Supplement: File S1 — Figures S1-S9. Figure S1. Schematic representation of repetitive DNA sequences present in the HPRT vectors used in this study. The location and length (bp) of each SINE/LINE fragment is based on the UCSC Genome Browser Database: Update 2006 (Nucleic Acids Res. 34:D590–D598, 2006). Figure S2. Impact of siRNA-mediated knockdown of DNA ligase I or IIIα on integration frequency. (A) The nucleotide sequence of LIG1 and LIG3 siRNA. These siRNAs were designed as reported previously (Nucleic Acids Res. 36: 3297–3310, 2008). (B) Western blot analysis for DNA ligase I and IIIα in siRNA-transfected Nalm-6 wild-type and LIG4-null cells. M, mock-transfected. (C, D) Integration frequency in wild-type and LIG4-null cells treated with LIG1 siRNA (C) or LIG3 siRNA (D). A non-targeting vector (pβactin-His; Nucleic Acids Res. 36: 6333–6342, 2008) was used for transfection. The integration frequency in mock-transfected wild-type cells was taken as 1, and the relative integration frequency was calculated. Figure S3. Structural features of gene-targeting vectors used for the analysis of integration frequency. (A) Fundamental structure of targeting vectors. In all the vectors, 5' and 3' arms flank the drug-resistance gene cassette (Puror), which is placed in the forward direction. (B) Structural features of the fourteen gene-targeting vectors used. Shown are the lengths of 5' and 3' arms and SINE/LINE sequences within each arm and the integration frequency. The length of SINE/LINE is based on the UCSC Genome Browser Database: Update 2006 (Nucleic Acids Res. 34:D590–D598, 2006). Figure S4. Integration frequency of targeting vector as a function of the length of repetitive DNA sequences. Integration frequencies of pHPRT8.9-Puro(+), pHPRT2.2-Puro(+), and twelve other gene-targeting vectors are shown as a function of the total length of SINE sequence (A), 5’-arm SINE length (B), 3’-arm SINE length (C), the total length of LINE sequence (D), 5’-arm LINE length (E), and 3’-arm LINE length (F). [file pone.0108236.s001.pdf]

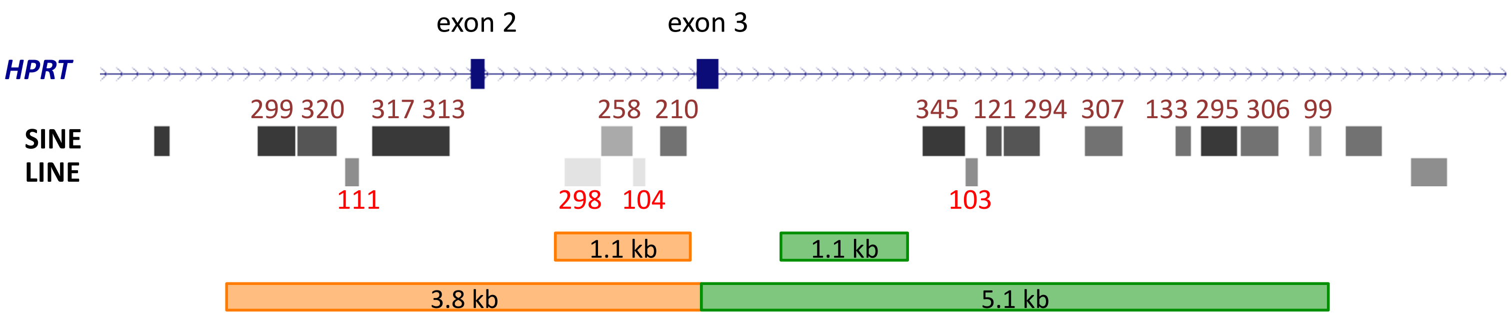

Ishii *et al*.

Figure S1.

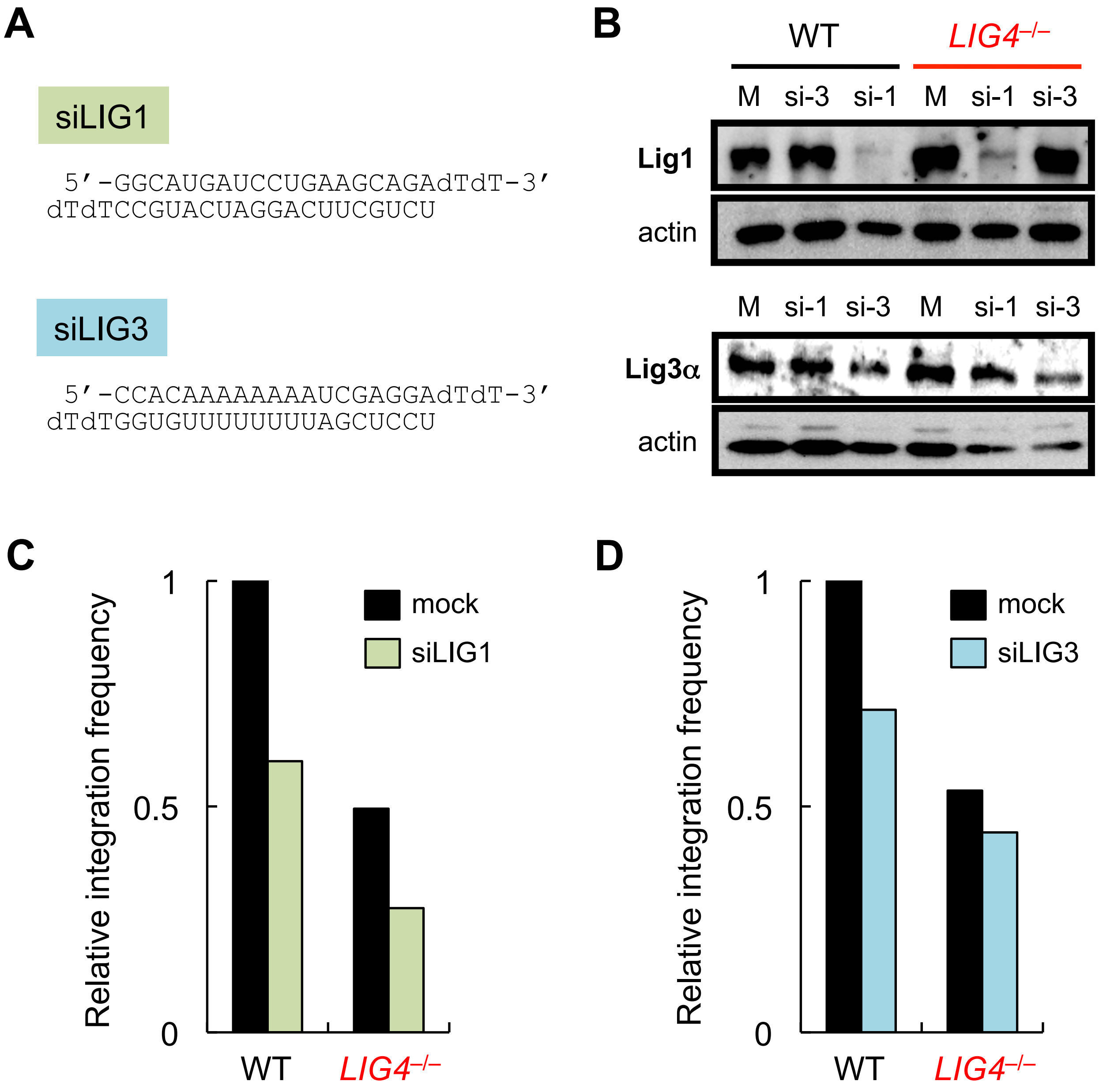

Ishii *et al.*
Figure S2.

A

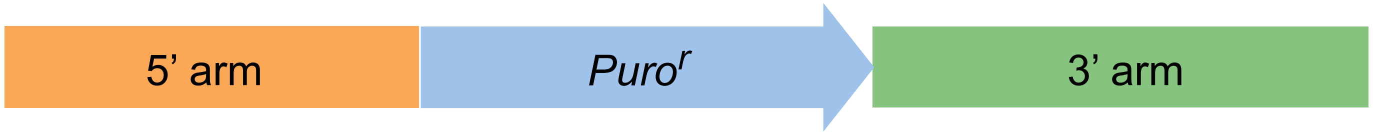

B

Locus	5' arm				3' arm				Integration frequency (x10 −4)
	Length (kb)	SINE (bp)	LINE (bp)	SINE+LINE (bp)	Length (kb)	SINE (bp)	LINE (bp)	SINE+LINE (bp)	
HPRT (2.2 kb)	1.1	468	402	870	1.1	0	0	0	0.7
ARTEMIS	1.0	481	0	481	2.4	601	0	601	0.7
APTX	2.6	1077	287	1364	2.3	519	556	1075	0.5
KU86	2.8	496	540	1036	2.3	0	290	290	1.4
MUS81	3.2	205	0	205	2.5	0	0	0	0.9
SIRT1	3.0	613	298	911	3.1	1463	0	1463	0.9
RAD54	2.6	1157	0	1157	3.5	1111	0	1111	2.3
CTIP	3.0	432	535	967	3.2	1214	646	1860	2.6
TOP2A	2.5	1204	138	1342	4.3	1698	83	1781	2.8
RAG1	2.8	295	695	990	4.0	211	94	305	2.4
TP53BP1	2.6	1298	336	1634	4.7	818	0	818	1.9
POLB	2.3	822	0	822	5.1	1535	541	2076	2.0
DROSHA	2.9	302	499	801	4.8	756	395	1151	1.5
HPRT (8.9 kb)	3.8	1717	513	2230	5.1	1900	103	2003	1.9

Ishii *et al.*

Figure S3.

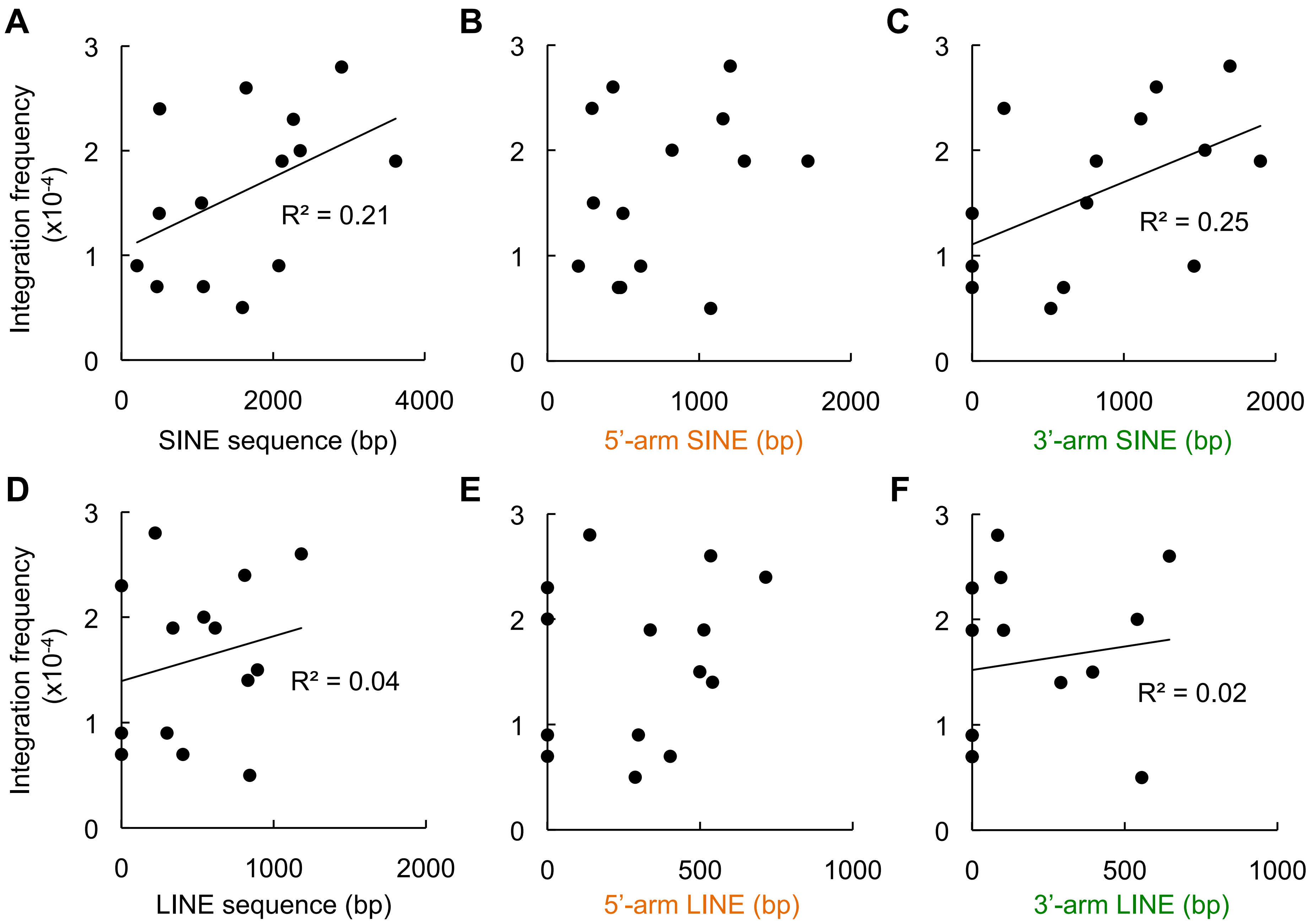

Ishii *et al.*

Figure S4.

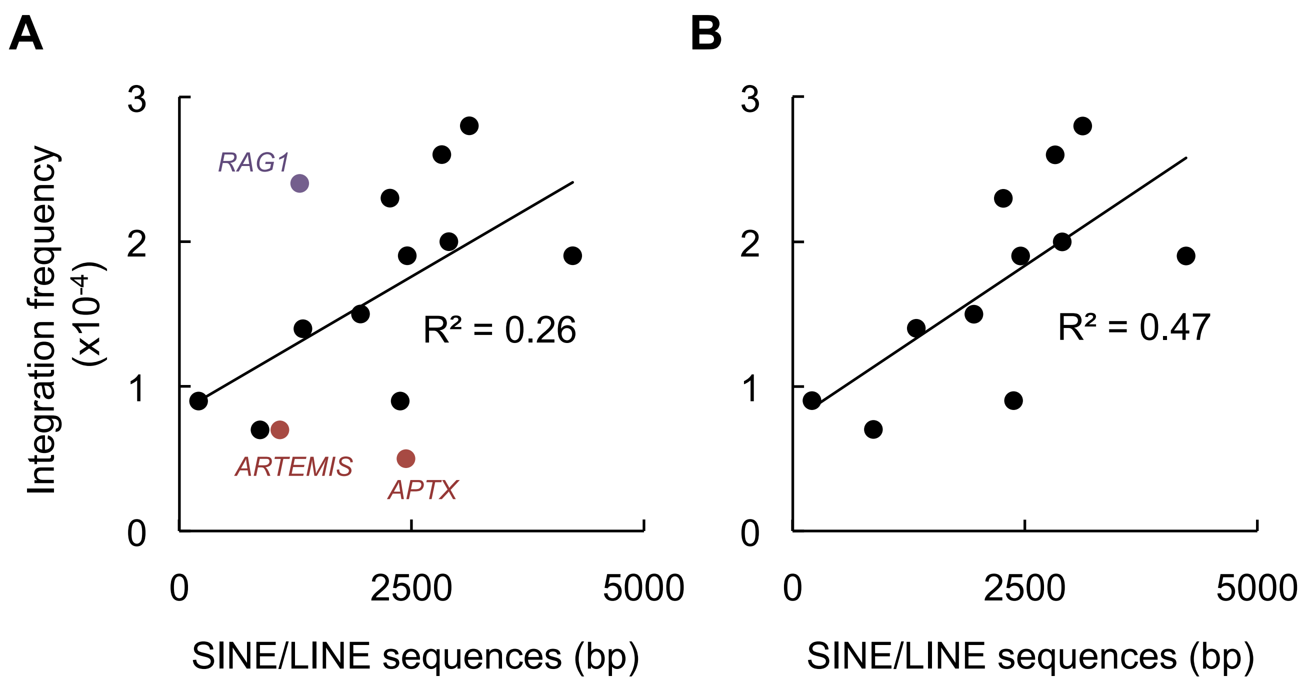

Ishii *et al*.
Figure S5.

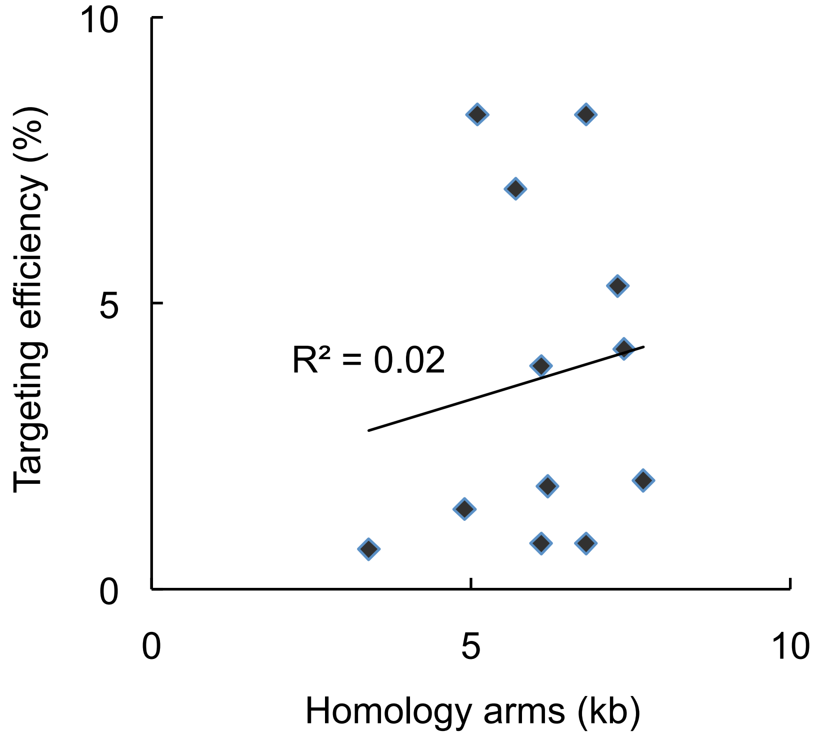

Ishii *et al*.

Figure S6.

Name	Sequence
2.2(+)-5F	5’-GGGGACAACTTTGTATAGAAAAGTTGGAGCTCTTCTCAAGCACTGGCTATGCATGT-3’
2.2(+)-5R	5’-GGGGACTGCTTTTTTGTACAAACTTGGAGCTCACCTCTTAGTCATTAAACTTCCACAC-3’
2.2(+)-3F	5’-GGGGACAGCTTTCTTGTACAAAGTGGGTCGACCAGAAACCACTTTTGATCCACAGTCT-3’
2.2(+)-3R	5’-GGGGACAACTTTGTATAATAAAGTTGGTCGACGATGTCATGCTTTTTATCAGTTGAGGAG-3’
2.2(-)-5F	5’-GGGGACAACTTTGTATAATAAAGTTGGAGCTCTTCTCAAGCACTGGCTATGCATGT-3’
2.2(-)-5R	5’-GGGGACAGCTTTCTTGTACAAAGTGGGAGCTCACCTCTTAGTCATTAAACTTCCACAC-3’
2.2(-)-3F	5’-GGGGACTGCTTTTTTGTACAAACTTGGTCGACCAGAAACCACTTTTGATCCACAGTCT-3’
2.2(-)-3R	5’-GGGGACAACTTTGTATAGAAAAGTTGGTCGACGATGTCATGCTTTTTATCAGTTGAGGAG-3’

Ishii *et al*.

Figure S7.

Gene	Arm	Direction	Sequence	Location
APTX	5' arm	Fwd	5'-GGGGACAACTTTGTATAGAAAAGTTGGACCTATGGCTTGCCATTGCCGAA-3'	Intron 2
		Rev	5'-GGGGACTGCTTTTTTGTACAAACTTGAACAGATCCACTAGCACGAGTCCT-3'	Intron 2
	3' arm	Fwd	5'-GGGGACAGCTTTCTTGTACAAAGTGGACTGTGACAGTAGTACTGTGCTGC-3'	Intron 6
		Rev	5'-GGGGACAACTTTGTATAATAAAGTTGCAATGCTTGAGGGAGGATTAACTG-3'	Intron 7
ARTEMIS	5' arm	Fwd	5'-GGGGACAACTTTGTATAGAAAAGTTGAGCATCTCCATTCTGTTATTTTCTA-3'	Intron 5
		Rev	5'-GGGGACTGCTTTTTTGTACAAACTTGTGGATCACAGAACGTAGTATCCA-3'	Exon 7
	3' arm	Fwd	5'-GGGGACAGCTTTCTTGTACAAAGTGGTTAGGCAGAGGAATATTTTCAGTGG-3'	Intron 9/Exon 10
		Rev	5'-GGGGACAACTTTGTATAATAAAGTTGTCAGTATTATGACTGGTCACCA-3'	Intron 11
RAD54	5' arm	Fwd	5'-GGGGACAACTTTGTATAGAAAAGTTGCACATTCTTCCTTACCAGTTATGC-3'	Intron 3
		Rev	5'-GGGGACTGCTTTTTTGTACAAACTTGTACCACAGACTTAGCCAACCTGAG-3'	Intron 3
	3' arm	Fwd	5'-GGGGACAGCTTTCTTGTACAAAGTGGGCCAGAGTCCAGAGTGCAAGCCAG-3'	Exon 7
		Rev	5'-GGGGACAACTTTGTATAATAAAGTTGAAAAGCGTTACTGGGAGGAAGATG-3'	Intron 8
TOP2A	5' arm	Fwd	5'-GGGGACAACTTTGTATAGAAAAGTTGAATGCTGCGGACAACAAACAAAGG-3'	Exon 4
		Rev	5'-GGGGACTGCTTTTTTGTACAAACTTGAGCTGTCCAAATATGAGAGCTGGG-3'	Exon 5
	3' arm	Fwd	5'-GGGGACAGCTTTCTTGTACAAAGTGGTGGAACTCAAGCCCTTCAATGGAG-3'	Exon 7
		Rev	5'-GGGGACAACTTTGTATAATAAAGTTGTACAATACCACAGCCAATGGCCTG-3'	Exon 11
53BP1	5' arm	Fwd	5'-GGGGACAACTTTGTATAGAAAAGTTGTTTCACTTGCTTGGGTCACGGTTC-3'	Intron 4
		Rev	5'-GGGGACTGCTTTTTTGTACAAACTTGTTATTCCACTGACATTCCCAGAACAC-3'	Exon 5
	3' arm	Fwd	5'-GGGGACAGCTTTCTTGTACAAAGTGGATTTAAATCCTGCAAGGTAAGCCGAACTTTCT-3'	Exon 7
		Rev	5'-GGGGACAACTTTGTATAATAAAGTTGGGAGTAGATCGGAAAGCATCAGGA-3'	Exon 10
RNASEN	5' arm	Fwd	5'-GGGGACAACTTTGTATAGAAAAGTTGTGTAGCTGATGACAAAGAAGGTGATGT-3'	Intron 5
		Rev	5'-GGGGACTGCTTTTTTGTACAAACTTGTTAGTCAGTTACCGCGGTTCAGGAACAACCGATA-3'	Exon 6
	3' arm	Fwd	5'-GGGGACAGCTTTCTTGTACAAAGTGGGGCCATGGCTGGTGTTCACTGAGTG-3'	Intron 7
		Rev	5'-GGGGACAACTTTGTATAATAAAGTTGGCCCAGAGCAATAACCGTTATGTCTG-3'	Intron 9
RAG1	5' arm	Fwd	5'-GGGGACAACTTTGTATAGAAAAGTTGAGGAGTGTAGATGTCTCTGAACATA-3'	Intron 1
		Rev	5'-GGGGACTGCTTTTTTGTACAAACTTGTGGCTGAGGTACCTGAGAACAATGA-3'	Intron 1/Exon 2
	3' arm	Fwd	5'-GGGGACAGCTTTCTTGTACAAAGTGGTTATGCTGGCAGATGAGTCTGACCACGA-3'	Exon 2
		Rev	5'-GGGGACAACTTTGTATAATAAAGTTGATAATGGTTTATTCCTCCCAACTAC-3'	Exon 2
MUS81	5' arm	Fwd	5'-GGGGACAACTTTGTATAGAAAAGTTGCTTCCGTTTAGTAGGAGCCGCACAA-3'	Exon 1
		Rev	5'-GGGGACTGCTTTTTTGTACAAACTTGGGCAAGGAGGGAAGCCTAGATCAGA-3'	Intron 8
	3' arm	Fwd	5'-GGGGACAGCTTTCTTGTACAAAGTGGTTGCTGGCTTTGCCAGGCTTCCCTG-3'	Intron 10
		Rev	5'-GGGGACAACTTTGTATAATAAAGTTGCAATGAGCCCTCTGCACTCCTAAGG-3'	Exon 16
CTIP	5' arm	Fwd	5'-GGGGACAACTTTGTATAGAAAAGTTGTACCTCAGTACTACTTCTGGGTCT-3'	Upstream of exon 1
		Rev	5'-GGGGACTGCTTTTTTGTACAAACTTGAGGTATCAAGTCGTCTTTGGACAG-3'	Exon 2
	3' arm	Fwd	5'-GGGGACAGCTTTCTTGTACAAAGTGGACAGCCTGCAGTCTTAGTAGAGTC-3'	Intron 2
		Rev	5'-GGGGACAACTTTGTATAATAAAGTTGTATTGACGAAGGCATGGCCCAAAC-3'	Intron 2
KU86	5' arm	Fwd	5'-GGGGACAACTTTGTATAGAAAAGTTGAGTGGTAGTTGTCTCTGAAGGGTC-3'	Intron 1
		Rev	5'-GGGGACTGCTTTTTTGTACAAACTTGCAGCTGCCTGGAAACAAAGTTCCA-3'	Intron 1/Exon 2
	3' arm	Fwd	5'-GGGGACAGCTTTCTTGTACAAAGTGGTAAGATGGATGCTTGTCTAGGCGG-3'	Intron 2
		Rev	5'-GGGGACAACTTTGTATAATAAAGTTGTCCATGCTCACGATTAGTGCATCC-3'	Exon 4
SIRT1	5' arm	Fwd	5'-GGGGACAACTTTGTATAGAAAAGTTGCTATGGCAGCAACAGAATGGCCCAG-3'	Intron 4
		Rev	5'-GGGGACTGCTTTTTTGTACAAACTTGCAGAGAGATGGCTGGAATTGTCCAGG-3'	Exon 5
	3' arm	Fwd	5'-GGGGACAGCTTTCTTGTACAAAGTGGGGGTCTTCCCTCAAAGTAAGACCAGT-3'	Exon 7
		Rev	5'-GGGGACAACTTTGTATAATAAAGTTGGAGGCACTTCATGGGGTATGGAACC-3'	Intron 7/Exon 8
POLB	5' arm	Fwd	5'-GGGGACAACTTTGTATAGAAAAGTTGCATCCTGGCCAACATGGTGAAACATC-3'	Intron 2
		Rev	5'-GGGGACTGCTTTTTTGTACAAACTTGAGGAGGATATACTGATGCTGAGAAC-3'	Intron 3
	3' arm	Fwd	5'-GGGGACAGCTTTCTTGTACAAAGTGGATGAGTAACCATGGCAGAGCCAGA-3'	Intron 5
		Rev	5'-GGGGACAACTTTGTATAATAAAGTTGGATGAAGCACCTATTCACAGCC-3'	Intron 7

Ishii *et al.*

Figure S8.

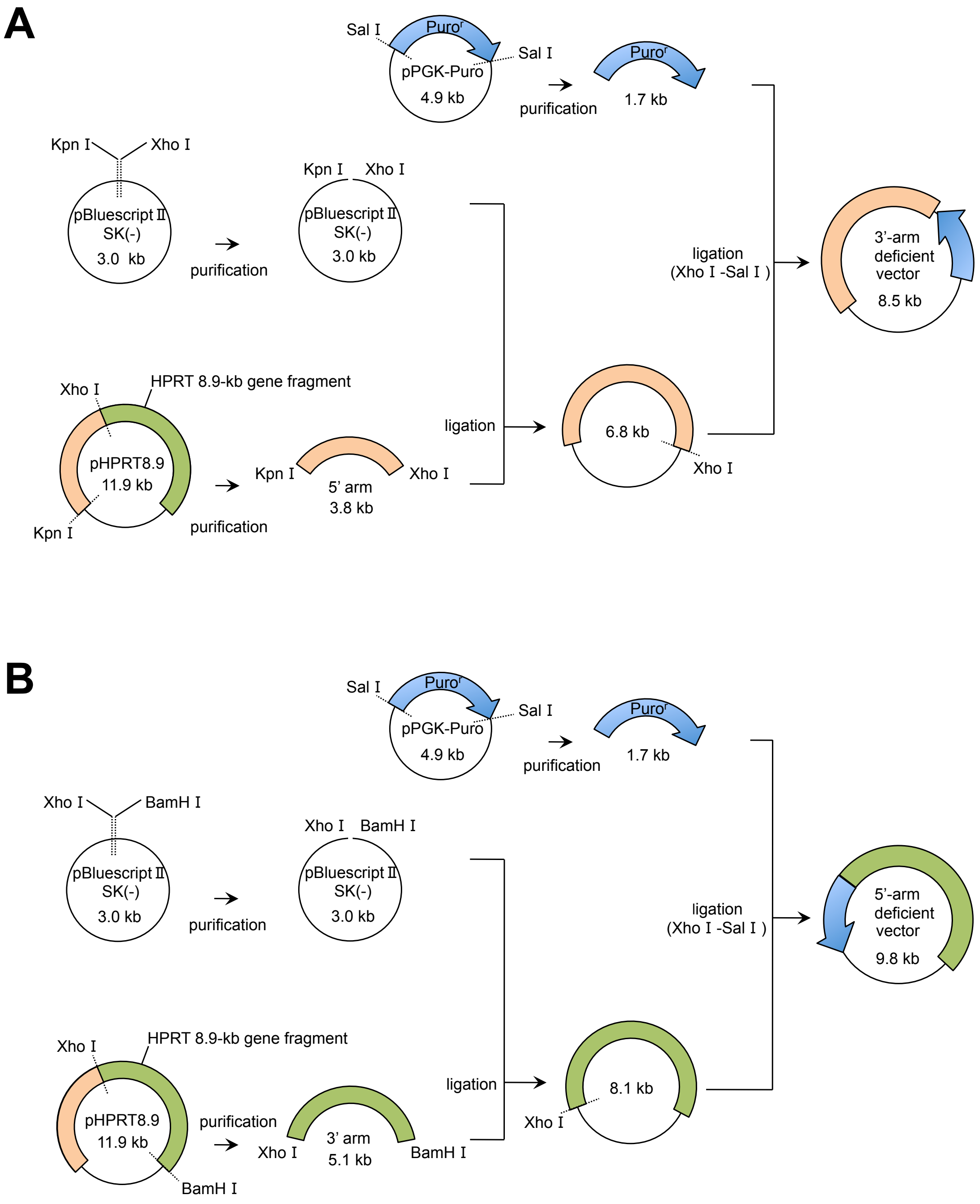

Ishii *et al*.
Figure S9.
